# Visualization, imaging and new preclinical diagnostics in radiation oncology

**DOI:** 10.1186/1748-717X-9-3

**Published:** 2014-01-03

**Authors:** Clemens C Cyran, Philipp M Paprottka, Michel Eisenblätter, Dirk A Clevert, Carsten Rist, Konstantin Nikolaou, Kirsten Lauber, Frederik Wenz, Daniel Hausmann, Maximilian F Reiser, Claus Belka, Maximilian Niyazi

**Affiliations:** 1 Department of Clinical Radiology, Laboratory of Experimental Radiology, University of Munich Hospitals, Campus Großhadern, Marchioninistraße 15, 81377 Munich, Germany; 2 Department of Radiation Oncology, University of Munich Hospitals, Campus Großhadern, Marchioninistraße 15, 81377 Munich, Germany; 3 IZKF Core Unit OPTI, Department of Radiology, University Hospital Muenster, Albert-Schweitzer-Campus 1, Muenster, Germany; 4 Comprehensive Cancer Imaging Centre, Division of Imaging Sciences & Biomedical Engineering, King’s College London, The Rayne Institute, 4th Floor Lambeth Wing, St. Thomas Hospital, London SE1 7EH, UK; 5 Department of Radiation Oncology, University Medical Centre Mannheim, University of Heidelberg, Theodor-Kutzer-Ufer 1-3, Mannheim 68167, Germany; 6 Institute of Clinical Radiology and Nuclear Medicine, University Medical Center Mannheim, Medical Faculty Mannheim, Heidelberg University, Mannheim, Germany

**Keywords:** Radiation oncology, Molecular imaging, Functional imaging, Preclinical models

## Abstract

Innovative strategies in cancer radiotherapy are stimulated by the growing knowledge on cellular and molecular tumor biology, tumor pathophysiology, and tumor microenvironment. In terms of tumor diagnostics and therapy monitoring, the reliable delineation of tumor boundaries and the assessment of tumor heterogeneity are increasingly complemented by the non-invasive characterization of functional and molecular processes, moving preclinical and clinical imaging from solely assessing tumor morphology towards the visualization of physiological and pathophysiological processes. Functional and molecular imaging techniques allow for the non-invasive characterization of tissues in vivo, using different modalities, including computed tomography (CT), magnetic resonance imaging (MRI), ultrasound, positron emission tomography (PET) and optical imaging (OI). With novel therapeutic concepts combining optimized radiotherapy with molecularly targeted agents focusing on tumor cell proliferation, angiogenesis, and cell death, the non-invasive assessment of tumor microcirculation and tissue water diffusion, together with strategies for imaging the mechanisms of cellular injury and repair is of particular interest. Characterizing the tumor microenvironment prior to and in response to irradiation will help to optimize the outcome of radiotherapy. These novel concepts of personalized multi-modal cancer therapy require careful pre-treatment stratification as well as a timely and efficient therapy monitoring to maximize patient benefit on an individual basis. Functional and molecular imaging techniques are key in this regard to open novel opportunities for exploring and understanding the underlying mechanisms with the perspective to optimize therapeutic concepts and translate them into a personalized form of radiotherapy in the near future.

## Introduction

The effective use of radiation for cancer treatment is closely linked to the optimal application of imaging for staging and tumor characterization. Therefore any improvement in the field of imaging will impact on radiation oncology per se. In a broader sense the term imaging may not only be used to cover aspects of patho-anatomical imaging but may also cover all relevant aspects of additional functional visualization. The growing knowledge on the pathophysiology of cancer and the associated paradigm shift in therapeutic concepts are moving preclinical and clinical imaging from exclusively assessing tumor morphology towards the visualization of physiological and pathophysiological processes on a molecular level. Functional and molecular imaging allows for the non-invasive characterization of tissues in vivo, and comprises techniques, such as computed tomography (CT), magnetic resonance imaging (MRI), ultrasound, positron emission tomography (PET) and optical imaging (OI). These novel imaging techniques have the potential for the visualization of functional tumor properties and the quantification of molecular pathways regulating the hallmarks of cancer [1]. As such, signaling pathways orchestrating proliferation, survival, angiogenesis, invasiveness, metastasis, and different types of cell death can be visualized either directly or indirectly via surrogate markers [2]. Imaging the mechanisms of cellular injury, repair, and cell death is of particular interest for characterizing the tumor microenvironment prior to and in response to irradiation, and hence for optimizing the outcome of radiotherapy (RT) [3].

RT is an established, highly effective cancer treatment option applied for definite, curative treatment as well as for palliative care. Together with surgery and/or chemotherapy it is an integral part of multimodality approaches. Novel therapeutic concepts include optimized radiotherapy in combination with molecularly targeted agents focusing on tumor cell proliferation, angiogenesis, and cell death [4]. Importantly, these concepts require predictive biomarkers in order to stratify tumors for the appropriate therapy according to their individual molecular profile as well as biomarkers for monitoring the therapeutic outcome on a non-invasive and serial basis *in vivo*. E.g. with regard to tumor heterogeneity, the non-invasive evaluation of cellular tumor properties, such as proliferation, using molecular imaging methods could be of great interest for radiotherapy planning, for the identification of highly proliferative tumor areas. Functional and molecular imaging techniques may be key in this regard, since they open novel and exciting opportunities for exploring the molecular mechanisms in radiation biology with the possibility to optimize therapeutic concepts and translate them into a personalized form of radiotherapy in future.

### Imaging modalities

#### ***Computed Tomography (CT)***

CT is one of the leading imaging modalities in medical imaging and standard-of-care in RT planning. Advantages include short examination times allowing for whole body imaging within seconds, broad availability of the technique as well as low costs. Additionally, CT offers high spatial resolution at the submillimeter level. In small animal imaging, morphologic micro CT has shown significant potential with benefits, including high throughput and superior resolution. Winkelmann and colleagues investigated micro CT in a bone metastasis model of prostate cancer in mice and found that bone micro CT was able to non-invasively follow the onset and progression of bone metastatic lesions as small as 300 μm in diameter [5]. Although radiation dose per micro CT scan approached 7–9 cGy, with six to nine micro CT examinations per mouse over a 7-week period, the applied radiation dose did not induce tumor stasis. However, the radiation dose applied by diagnostic micro CT has to be taken into account, particularly, when investigating micro CT for RT planning in small animal tumor models. A study by Boll and colleagues [6] investigated a dedicated alkaline earth metal-based nanoparticulate contrast agent for micro CT imaging of liver metastases in a colon carcinoma metastasis model in mice and reported that liver metastases as small as 300 μm were detectable after a single injection. The authors concluded that the investigated nanoparticulate contrast agent is suitable to compensate for the limited soft tissue contrast of unenhanced micro CT and allows for high resolution and high soft tissue contrast imaging of tumors in small animal models. Therefore, micro CT enhanced with dedicated contrast media may be of particular interest for the delineation and non-invasive characterization of tumors before and during radiotherapy in the preclinical setting.

Recent studies also support the value of perfusion CT as a functional imaging method in oncology [7]. Perfusion imaging techniques based on CT, MRI and ultrasound have been applied for the non-invasive quantification of functional parameters of tissue microcirculation [8-11]. It has also been shown that dynamic contrast-enhanced CT (DCE-CT) allows for the assessment of pathologically increased tissue perfusion, blood volume and permeability [12], reflecting typical features of angiogenically active tissues, such as tumors. Surrogate parameters of tumor microcirculation assessed by DCE-CT have the potential to predict response to chemotherapy or irradiation in various cancers, e.g. cancers of the head and neck, lung, and rectum [13-15]. In anti-angiogenic tumor therapy, DCE-CT has shown its applicability for early assessment of the therapeutic effect on tumor vascularization, identifying treatment responders from non-responders, and optimizing personalized molecular therapies on an individualized patient basis.

Dual-energy CT (DECT) offers high soft tissue contrast and a clear differentiation between soft tissue, iodine contrast and bone. Dual source DECT can provide iodine maps which reflect iodine content in a tissue of interest and which have been demonstrated to show good correspondence to perfusion images in the lung and heart [16,17]. In an experimental study of a VX2-rabbit model of liver cancer Zhang and colleagues reported that DECT iodine maps correlated well with multiparametric perfusion CT measurements for monitoring tumor angiogenesis with a significantly lower effective radiation dose. It was concluded that DECT might have the potential for serially monitoring angiogenesis in solid tumors with a significant reduction in radiation dose compared to perfusion CT techniques [18]. This technique may be of particular interest for serially monitoring tumor heterogeneity and angiogenesis in the planning and monitoring of RT combined with anti-angiogenic agents.

### Magnetic Resonance Imaging (MRI)

Based on strong magnetic fields (clinically between 1.5 and 3 Tesla, human research scanners up to 7 Tesla) MRI is able to provide superior soft tissue contrast and high spatial resolution without the application of ionizing radiation. Besides its morphologic capabilities useful for clinical staging and RT planning purposes [19,20], MRI is increasingly developed for functional and molecular imaging methods, among them perfusion and diffusion imaging as well as MR spectroscopy and molecular MRI. Perfusion MRI can be applied to quantify functional parameters of tissue microcirculation, which have been shown to reflect tissue properties such as vitality, angiogenesis and proliferation [21]. Novel imaging methods such as ^23^Natrium MRI have been proposed as a potential imaging biomarker for the assessment of tumor viability and the evaluation of therapy response in cancer patients [22].

#### ***Magnetic Resonance Proton Spectroscopy (MRS)***

MR spectroscopy uses selective radiofrequency pulses for the investigation of the molecular composition of tissues [23,24]. The Fourier transformation of the acquired signal generates a defined spectrum allowing for the discrimination of different metabolites in the investigated tissue, which may be pathognomonic for certain underlying pathologies. Metabolites detected in tumor tissues include choline-containing compounds, creatine, glutamate, lactate, N-acetyl aspartate (NAA), myoinositol (mI) and taurine [25]. The concentration of each of these metabolites can be mapped on spectroscopic images with a voxel size of 0.7-1 cm^3^. NAA is predominantly a neuronal marker and decreases associated with neuronal damage and dysfunction [25]. Choline is associated with cell membrane synthesis as well as increased metabolic turnover and is elevated in tumors and inflammatory processes [25]. Creatine has been shown to be a marker of energy metabolism in the brain [26], while mI was confirmed as a glial cell marker and has been used as an indicator of myelin breakdown [27]. In glioblastomas increased levels of creatine and choline as well as a lowered level of N-acetyl aspartate were found [28]. Additionally, MRS can be applied for pre-operative staging of gliomas [29] and for monitoring tissue pH and temperature [30]. With regard to radiation therapy, MRS may be a sensitive tool for monitoring radiation-induced changes in tumors based on the acquired spectrum of metabolites. MRS has also been postulated to be of particular interest in focal dose escalation in prostate cancer patients [31]. Significant technical challenges for clinical translation remain particularly with regard to reproducibility in the quantification of chemical metabolites in tumors as well as impeded data quality due to local-field inhomogeneities caused by healthy tissue adjacent to the tumor [26].

#### ***MR perfusion***

MR perfusion represents one of the most promising methods of functional MR imaging. Perfusion imaging is defined as the *in vivo* assessment and quantification of microcirculatory parameters in different tissues, which may allow for the characterization of an underlying pathology. Depending on the imaging protocol and kinetic models applied for data analysis different parameters of microcirculation, such as plasma flow, extraction fraction, or relative plasma volume, can be assessed reflecting tissue properties, such as tissue perfusion, endothelial permeability, and tissue vascularity [32], *in vivo*. Technically, MR perfusion imaging can be performed by dynamic contrast-enhanced imaging (DCE), dynamic susceptibility contrast imaging (DSC), and arterial spin labeling (ASL) techniques. The most common method for perfusion imaging, however, is dynamic contrast-enhanced imaging.

**Figure 1:**
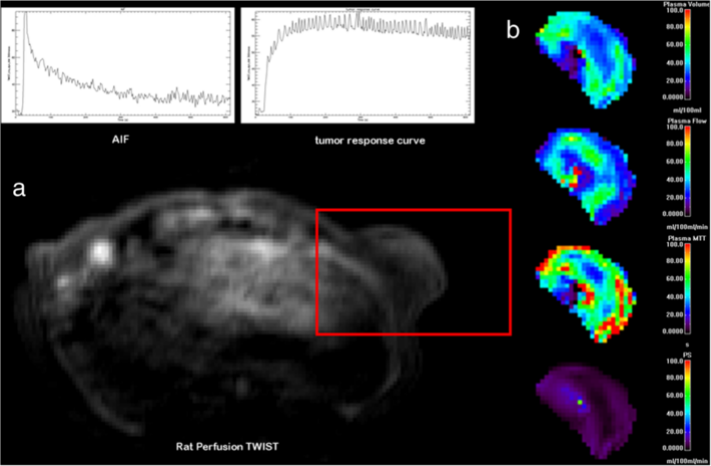
**Representative axial T1 weighted TWIST image of an athymic rat bearing a subcutaneous colon carcinoma xenograft over the left flank. (a)** Representative axial T1 weighted TWIST image of an athymic rat bearing a subcutaneous colon carcinoma xenograft over the left flank with the arterial input function (AIF) measured in the inferior vena cava as well as the signal enhancement curve over the tumor. **(b)** Representative quantitative parameter maps depicting different parameters of tumor microcirculation (from top to bottom): plasma volume (%), plasma flow (ml/100 ml/min), plasma mean transit time (s), permeability surface area product (ml/100 ml/min).

Methodologically, DCE imaging monitors signal enhancement before, during and after intravenous injection of a paramagnetic contrast agent applying T1-weighted sequences with high temporal and spatial resolution [21]. The resulting signal-intensity vs. time curve can be analyzed to yield different quantitative and semi-quantitative parameters, for example maximum signal enhancement, time-to-peak, maximum slope or area under the curve. These semi-quantitative parameters describe important aspects of contrast media kinetics and can be routinely assessed with high robustness. However, physiological interpretation of these semi-quantitative parameters is often difficult [33] and factors like acquisition time, temporal resolution, sequence parameters, contrast media dose, and bolus velocity greatly influence semi-quantitative parameters. As a consequence, semi-quantitative parameters are only of limited use for follow-up measurements and multicenter studies. As demonstrated in Figure 1, DCE-MRI can also be applied for the assessment of quantitative parameters of tissue microcirculation, such as plasma flow (ml/min/100 ml), extraction flow (ml/min/100 ml), and plasma volume (%) [34]. These parameters are considered to be physiologically distinct with better suitability for longitudinal and multicenter studies, but strongly depend on standardized imaging and analysis protocols. Depending on the kinetic profile of the contrast medium, investigated tissue of interest and acquisition technique a range of kinetic models is available for the analysis of the kinetic data, e.g. the Patlak or the Tofts model [35]. The best model to fit the data can be evaluated using the Akaike’s information criterion, which can be applied to support model selection for the mathematical description of tracer kinetics [36]. Clinically, MR perfusion imaging has great potential as a functional imaging method in oncology for the assessment of tumor vitality, angiogenesis, and tumor heterogeneity. In e.g. prostate cancer, it can be used after radical prostatectomy to detect local recurrence without an endorectal coil [37], for therapy response imaging [38] as well as for the determination of extracapsular extension [39]. However, standardization of acquisition protocols and data analysis remain major obstacles towards broader establishment of the technique in clinical routine [21].

#### ***MR diffusion***

**Figure 2:**
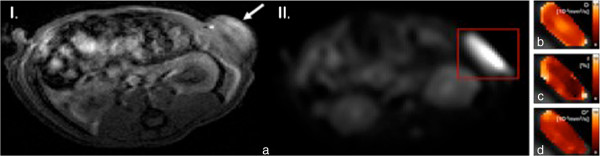
**Representative morphological and diffusion-weighted images of an athymic rat bearing a subcutaneous HNSCC (head and neck squamous cell carcinoma) over the left lateral flank. (I.)** T1-weighted flash 3D morphological image of an athymic rat bearing a subcutaneous HNSCC (head and neck squamous cell carcinoma) over the left lateral flank for anatomical correlation (white arrow). **(II.)** Example of a voxel-wise analysis of diffusion weighted images using the bi-exponential IVIM-model. Initial T2-weighted image is shown in **(a)**, **(b)**-**(c)** depict the calculated parameter maps for tissue diffusivity D **(b)**, perfusion fraction f **(c)** and pseudo diffusivity D*(d). Reduced values for f towards the center of the tumor indicate a local decrease in perfusion.

MR diffusion imaging is another major technique of functional MR imaging and is already routinely applied in neuroradiologic imaging protocols. In MR diffusion imaging, gradient pulses generate a spatially varying magnetic field with resulting phase differences of the MR signal, which are caused by the random motion of water molecules in the tissue (Brownian motion). Diagnostic applications of MR diffusion imaging include ischemia [40], tumor diagnosis and characterization [41], multiple sclerosis [42], therapy response assessment [38] as well as fiber tracking using diffusion tensor imaging (DTI). Recent studies have also investigated the potential of diffusion imaging for therapy monitoring [43] and the assessment of biomarkers in oncology with promising results [41]. In this context, Somford and colleagues successfully investigated DWI for the identification of high-grade prostate carcinoma in patients with a Gleason score ≥ 3 + 3 = 6 after TRUS-guided biopsy and concluded that DWI is able to predict the presence of high-grade tumor with significant relevance for subsequent treatment decisions. In a study of 73 patients with prostate cancer, Ueno et al. found that diffusion-weighted MRI with ultra high b-values (b = 2000 s/mm) is superior compared to the use of high b-values (b = 1000 s/mm) for prostate cancer detection, validated by histopathology following radical prostatectomy [44]. These ultra-high b-values may be assessable by computed DW MRI, as shown in a study by Blackledge and co-workers, who investigated DW MR imaging in 10 oncologic patients with b values of 0 and 900 sec/mm, subsequently generating images with computed b values of 1500 and 2000 sec/mm. They found that images with a computed b value of 2000 sec/mm resulted in higher overall diagnostic sensitivity and specificity compared to images with an acquired b value of 900 sec/mm [45]. A novel application of MR diffusion is intravoxel incoherent motion (IVIM) imaging, which allows to derive quantitative parameters that reflect tissue microcapillary perfusion and tissue diffusivity [46]. A bi-exponential model is fitted to diffusion-weighted data, to quantify the measured signal attenuation as a function of diffusion (b-value). Perfusion information is extracted from the initial signal attenuation at low b-values between 0 and ca. 150 s/mm^3^[46]. Figure 2 shows a representative example of a subcutaneous tumor xenograft of head and neck squamous cell carcinoma in rats imaged by diffusion-weighted MRI with voxel-wise analysis using the bi-exponential IVIM model.

Several recent studies have also discussed a possibly enhanced diagnostic value of multiparametric MRI combining morphologic and functional information from perfusion and diffusion parameters for the non-invasive characterization and differentiation of tumors [47]. The recently published guidelines of the European Society of Urogenital Radiology (ESUR) describe the application of multiparametric MRI for the detection and staging of prostate cancer, a separate protocol for node and bone imaging as well as a standardized reporting system (PI-RADS), analogue to breast imaging [48].

#### ***Molecular MRI (mMRI)***

mMRI applies targeted, gadolinium- or iron-based contrast agents of different designs, which allow for the dedicated depiction of molecular processes *in vivo* using antibodies, peptides or peptidomimetics [49,50]. Based on Gadolinium (Gd) or iron, these contrast agents cause a shortening of the T1- (Gd) or the T2/T2* time (iron) and lead to a change in tissue contrast. To achieve highly specific binding properties, Gd-chelates or nanoparticles are conjugated to antibodies, peptides or peptidomimetics for the dedicated *in vivo* visualization of molecular processes [50]. Serres and co-workers developed a targeted MRI contrast agent based on iron oxides that enables imaging of endothelial vascular cell adhesion molecule-1 (VCAM-1), which is known to be up-regulated on vessels of cerebral metastases [51]. They investigated whether MRI enhanced with the targeted anti-VCAM-1 microparticles of iron oxide (anti-VCAM-1 MPIO) would be able to depict up-regulated VCAM-1 in a model of human breast carcinoma cerebral metastasis in mice and if early detection of these metastases would be feasible. The results indicated that by use of the VCAM-1 targeted MRI contrast agent, it is possible to detect brain metastases substantially earlier than with the established gadolinium-based small molecular contrast media and concluded that this approach represents a highly sensitive method for the early detection of brain metastases with the potential for clinical translation [51]. Recently, enzymatic reporter systems for the non-invasive investigation of gene expression patterns detectable by MRI have been investigated combining the relatively high spatial and temporal resolution of MRI with the ability of each genetically-expressed enzyme to generate many MRI-detectable product molecules [52]. Currently, most of these molecular MR contrast agents are experimental and not approved for human use. Particularly concerns of potential immunogenicity and incomplete bio-elimination of targeted MR contrast agents hamper clinical translation.

#### ***Hyperpolarized MRS***

Compared to the currently established proton-based MR imaging, other nuclei like ^3^He, ^129^Xe or ^13^C have lower occurrence in the human body. If these alternative nuclei were used for the generation of the radiofrequency signal in MRI, the resulting signal-to-noise ratio (SNR) would be quite low. By means of hyperpolarization, however, it is possible to excite specific nuclei, thereby potentiating their MR signal to achieve a better SNR. ^3^He und ^129^Xe can be polarized by optical pumping, while ^13^C can be polarized using parahydrogen and dynamic polarization [53]. Different studies have investigated ^13^C in MR angiography- und perfusion studies as well as ^129^Xe for lung imaging [54,55]. Experimental, hyperpolarized MR contrast agents such as ^13^C-urea do not alter relaxation time, as established Gd- or iron based MR contrast media, but resemble in their function radioactive tracers, with the hyperpolarized nuclei representing the basis for the MR signal.

To date, ^13^C pyruvate has been the most widely used hyperpolarized substrate for MRS, which has also been applied for tumor response monitoring [56], and was the first to be used in a clinical trial of the technique [57]. In a study investigating the effects of the mTOR inhibitor everolimus on a highly invasive orthotopic glioblastoma model in rats. Chaumeil and colleagues demonstrated that hyperpolarized ^13^C MRS can be used on a clinical MR system to monitor early metabolic response by means of measurement of the HP lactate-to-pyruvate ratios [58]. Similarly, Day et al. showed the applicability of hyperpolarized ^13^C pyruvate MRS for the detection of treatment response 72 h following a whole brain irradiation with 15Gy in a rat glioma model [59]. Golman and colleagues investigated ^13^C pyruvate in P22 tumors in rats for the non-invasive imaging of the anaerobic glycolysis of the injected pyruvate to alanine and lactate, analogue to imaging of aerobic glycolysis with ^18^ F-FDG-PET [60]. Further perspectives of hyperpolarized MRI were discussed in a paper of Mansson et al. who were able to show that the signal of ^13^C nuclei varies depending on the hosting molecule, which could allow for refined discrimination of different ^13^C-containing molecules. This could be an advantage over radionuclide-based imaging modalities such as PET and SPECT [61]. Main problems of the still experimental hyperpolarization MRI include the very high costs as well as the rapid decline of the hyperpolarization, which allow only for a very short interval between application and imaging [62].

### Ultrasound

In recent years, ultrasound has undergone significant technological advancement with an evolution from a simple morphology-based gray-scale image to a multiparametric high-resolution real-time imaging system. Major developments include the introduction of functional imaging options including sophisticated Doppler ultrasound, contrast-enhanced ultrasound (CEUS), and elastography for the non-invasive characterization of tissues with significantly improved spatial and temporal resolution. The development of gas-filled blood-pool microbubble contrast agents has significantly enhanced clinical and pre-clinical research applications with particular regard to the *in vivo* characterization of tissue microcirculation in a semi-quantitative and quantitative manner [63]. Novel targeted microbubble contrast agents available for research purposes open the door for a molecular evaluation of tissues, e.g. by selectively binding to vascular endothelial growth factor receptor (VEGFR-2) [64-68] and a possible theranostics application linked to high intensity focused ultrasound (HIFU) which may be used in recurrent prostate cancer [69] and the microbubble-assisted delivery of drugs and genes [70].

Clinically, in recent years image-guided radiotherapy has been a major issue in research and development for (mage-guided radiotherapy (IGRT) and may be regarded as standard-of-care, especially in high-precision radiotherapy. One newly developed system is based on ultrasound – the Clarity 3D™ ultrasound system (Elekta, Stockholm, Sweden) is designed to track exemplarily the prostatic gland and adjacent organs-at-risk in order to minimize setup errors caused by organ motion, displacements and different filling states [71]. Furthermore, ultrasound has great potential to be established as a sensor for intrafractional movement as the tumor or organ motion can be tracked online as compared to a static cone beam CT (pre or post application of the individual fraction), e.g. in liver cancer/metastases or prostate cancer [72]. This in turn allows for an early detection of significant deviations and could in principle be used for real tumor tracking during irradiation.

#### ***Contrast-enhanced sonography (CEUS)***

**Figure 3:**
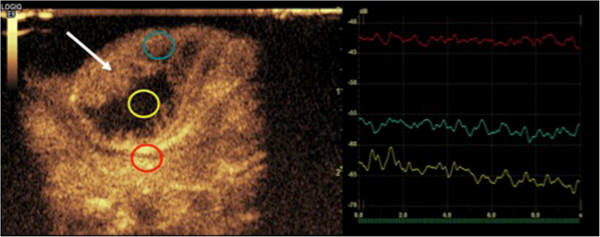
**Contrast-enhanced ultrasound (CEUS) with intravascular SF**_**6**_**-filled microbubbles: online quantification of hypervascular tumor tissue (turquoise ROI), tumor necrosis (yellow ROI) and feeding vessels (red ROI).**

In CEUS, gas-filled microbubbles are injected intravenously, thereby creating a multitude of small interfaces with high echogenicity. After destruction, the gas (e.g. SF_6_) is eliminated over the lungs within minutes and phospholipid membranes will be endogenously metabolized. The diameter of most microbubbles ranges between 2–10 μm, quite similar to the diameter of erythrocytes. Contrary to conventional small molecular CT- and MRI- contrast media, the microbubbles do not extravasate into the interstitial space, remain intravascular and therefore belong to the class of blood pool contrast media [73,74]. Microbubbles oscillate and vibrate resulting in a continuous improvement of gray scale contrast. With the development of high-frequency linear ultrasound transducers (>20 MHz) ever-smaller structures can be examined by CEUS with superior temporal and spatial resolution (Figure 3).

**Figure 4:**
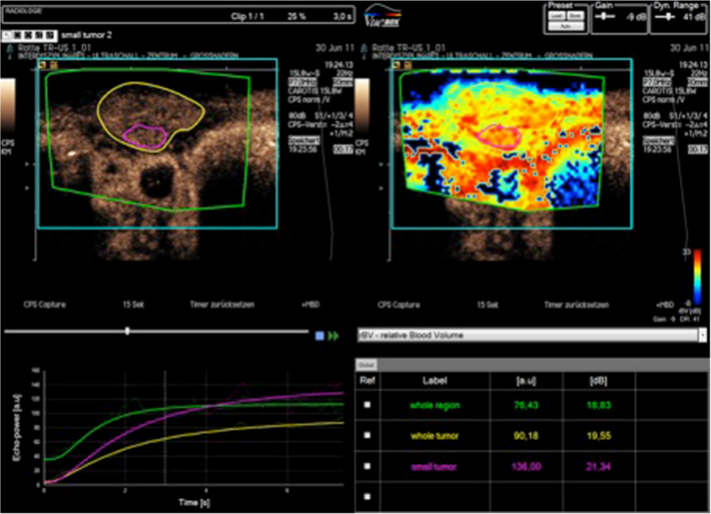
**Offline absolute quantification of perfusion of the whole tumor and hypervascular tumor parts using the flash replenishment method [**[89]**].** ROIs can be drawn in different parameter maps, allowing a simpler and more standardized analysis of the digitally stored video sequences.

For the most part CEUS has been applied for investigations of tumors in parenchymatous organs and in pathologies of the vascular system [75-83], where CEUS can be applied as a functional imaging method for the assessment of tissue microcirculation in healthy and malignant tissues. CEUS perfusion imaging may be particularly attractive for monitoring novel, molecular therapies in oncology mainly targeting tumor angiogenesis [84]. Paprottka and co-workers showed in an experimental study that CEUS allows for superior assessment of tumor perfusion compared to color-coded duplex ultrasound and power Doppler [84]. Other preclinical studies demonstrated that parameters of tumor microcirculation assessed by CEUS may be applicable as imaging biomarkers of tumor angiogenic activity and may have the potential to be used as non-invasive biomarkers of tumor responses under anti-angiogenic therapy (Figure 4) [85,86]. Radiotherapeutic applications of CEUS include treatment guidance in prostate brachytherapy planning [87] and it has a potential role in monitoring of liver metastases after stereotactic radiosurgery [88].

Apart from non-targeted, blood-pool ultrasound contrast media, targeted microbubbles have been developed as a molecular imaging technique by attaching specific ligands to the coating of gas-filled microbubbles. These targeted microbubbles can be applied for the non-invasive characterization of molecular tissue properties *in* vivo. As ultrasound microbubble contrast media remains intravascularly after intravenous injection, molecular targets have to be located on the luminal surface of vascular endothelium. Targeted microbubbles have been conjugated to ligands specific for highly expressed molecular markers of tumor angiogenesis such as VEGFR-2 and α_v_β_3_-integrin [64,65,68,70] to allow for the assessment of tumor angiogenic activity and for monitoring anti-angiogenic therapies in preclinical tumor models [90-92]. Together with potential application in theranostics multiparametric, contrast-enhanced ultrasound has developed to a high-potential tool for research and patient care combining high sensitivity, real-time morphological imaging, with functional and molecular imaging options with a lack of ionizing radiation and at comparably low costs [93].

#### ***Elastography***

**Figure 5:**
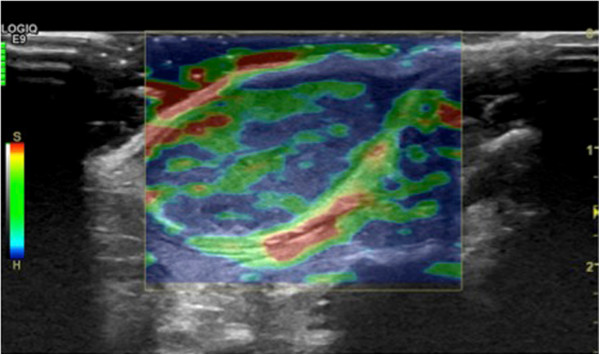
**Ultrasound elastography (blue: hard tissue, green: soft tissue): Central tumor necrosis is encoded in green.** Due to the immediate vicinity of subcutaneous tissues to the transducer, subcutaneous tumor parts under compression are also coded in green. Further developments investigate elastography without manual compression to minimize these artifacts.

**Figure 6:**
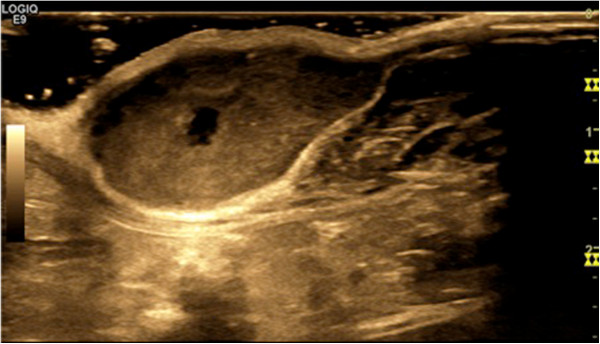
**Gray-scale ultrasound for the evaluation of tumor heterogeneity.** The extent of central tumor necrosis is significantly underestimated by gray-scale ultrasound compared to elastography.

Tissue elastography complements the conventional B-image, color Doppler, and CEUS in the assessment of pathologies [94,95]. Ultrasound elastography bridges the gap between modern state-of-the-art ultrasound and one of the most ancient examination techniques in medicine – palpation. Tissue elasticity is frequently altered in the presence of inflammation or malignancy and can be detected by compression elastography on the basis of compression and release [96]. Malignancies frequently exhibit higher tissue stiffness due to high rates of cell proliferation and densely packed cells. Thus, they appear less elastic in ultrasound elastography. By means of the combined autocorrelation method, elastic tissue properties of different tissues can be assessed (Figure 5). Application of light pressure on the tissue with the ultrasound probe and the subsequent release aid to assess relative stiffness of the investigated tissue and differences can be visualized, either in gray scale or color coded, parallel, or merged with a conventional b-image (Figure 6). Several studies investigated ultrasound elastography in mammography as well as in liver and prostate imaging and found that elastic tissue properties contribute valuable information, which is not discriminable in conventional ultrasound [97-101]. In an animal study elastography was applied to monitor ethanol injections for the treatment of liver tumors, and the authors concluded that elastography added significant information compared to the conventional b-image [102,103]. After adjuvant radiotherapy of breast cancer, elastography can be used to quantify the extent of lymph edema [104].

### Optical imaging

Optical imaging employs light for the assessment of functional and molecular tissue information. This light can either originate from administered, elicited fluorescent tracers or – as bioluminescence – from genetically modified cells [105]. Upon excitation with externally applied light of the proper wavelength, fluorescent tracers emit light with a higher wavelength that can be detected by a CCD (charged-coupled device) camera. Different forms of tracers have been described. For visualization of blood flow, unspecific, blood-flow distributing agents can be used, much resembling established contrast agents for x-ray computed tomography or magnetic resonance tomography [106]. For visualization of molecular processes, targeted probes have been designed, typically consisting of a fluorescent dye (e.g. Cy 5.5) and a binding moiety – e.g. an antibody or smaller peptide with binding specificity for the target of interest. To minimize tissue absorption and scattering of the emitted light, the optimal spectral range for *in vivo* applications has been defined as the near-infrared optical window (wavelengths 650–900 nm), with lowest tissue absorbance for hemoglobin, water and lipids. In bioluminescence, an *in vivo* enzymatic reaction is responsible for the emission of light. The most common enzymatic tool is the firefly luciferase system, where *D*-luciferine is oxidized using ATP (adenosine-tri-phosphate) and oxygen in a two-step mechanism. The resulting emission of yellow-green light at 575 nm can be employed to visualize luciferase-expressing cells *in vivo* following intravascular injection of D-luciferine, the substrate of firefly luciferase, or after providing D-luciferine in the drinking water. Advantages of bioluminescence imaging include (1) an exquisite imaging sensitivity due to a high signal-to-noise ratio caused by the lack of bioluminescence background signal (in mammals) and (2) the luciferase system does not require excitation light from outside to be activated. Disadvantages include the need for cell transfection with the luciferase reporter genes, substrate injection (*D*-luciferine), and the poor spatial resolution of bioluminescence imaging compared to tracer-mediated fluorescence optical imaging. In recent years, many studies have investigated the luciferase bioluminescence assay to visualize a wide array of molecular pathways for the non-invasive characterization of the tumor microenvironment as useful tools in radiation and cancer biology research.

**Figure 7:**
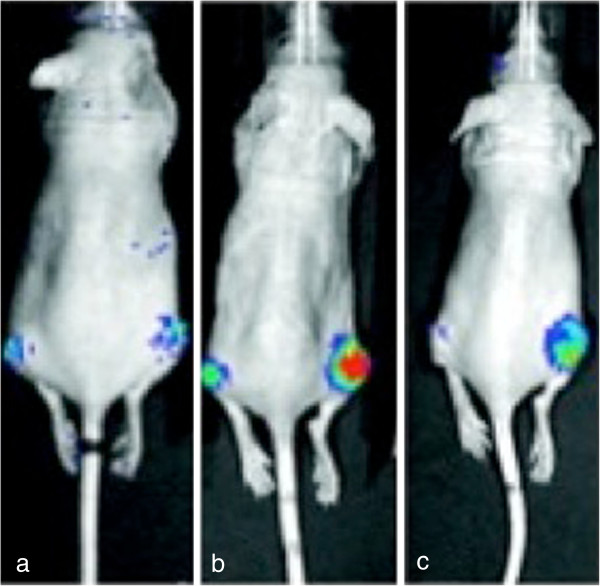
**Mice implanted with EGFR-luc transfected H322 tumors over the right flank. EGFR activation during radiotherapy (B, C; 3x6Gy) induces consecutive Luciferase expression and thus detectable bioluminescence upon luciferine injection.** Mice implanted into the right flank with EGFR-luc transfected H322 tumors. EGFR activation during radiotherapy (**B, C**; 3x6Gy) induces consecutive Luciferase expression and thus detectable Bioluminescence upon Luciferine injection. Changes to the EGFR activation by EGFR inhibition with e.g. Gefitinib **(C)** result in detectable alterations of the signal. Non-treated tumors **(A)** do not exhibit a specific signal. Adapted by permission from the American Association for Cancer Research: Li et al., Noninvasive imaging and quantification of epidermal growth factor receptor kinase activation in vivo. Cancer Research, 2008, 68, 4990–7. [109].

Bioluminescence optical imaging using firefly luciferase has been applied to image an array of biological pathways and cellular processes relevant for novel approaches in radiation and molecular cancer therapy [107]. In a recent study, Li and colleagues developed an imaging system for non-invasive quantification of epidermal growth factor receptor (EGFR) activation *in vivo* based on the bi-fragment luciferase reconstitution system. Epidermal growth factor and its receptor are part of a key-signaling cascade responsible for the initiation and growth of malignancies. Li and colleagues fused the EGF receptor and its interacting partner proteins growth factor receptor binding protein 2 (grb2), and Src homology 2 domain-containing protein (shc) to the aminoterminal and the carboxyterminal fragments of the firefly luciferase, respectively. In this system, firefly luciferase is only enzymatically active when the two parts of the protein are brought together, hence when EGFR, and grb2 or EGFR and shc are interacting and the signaling cascade is active. With the help of this system EGF-induced as well as radiation-induced pathway activation could be convincingly measured in vitro and in vivo (Figure 7) [108]. Moreover, this system was employed to visualize hyperthermia-induced EGFR activation in tumor cells and the potential mechanisms involved [109]. In an analogue study, Li and colleagues utilized their split-luciferase system in order to quantitatively assess DNA double strand breaks and their repair [110]. They fused the N- and C-terminal fragments of firefly luciferase with H2AX and MDC1, two proteins, which at the sites of DNA double strand breaks physically interact with each other. Hence, upon generation of DNA double strand breaks, the two luciferase fragments are brought together and luciferase activity can be detected at the site of damage. Since DNA double strand breaks and the mechanisms of their repair are of crucial interest in the context of ionizing radiation, this imaging system is of specific relevance for monitoring the effect of radiotherapy in tumor tissue.

The study of Backer and colleagues exemplary illustrates strengths and drawbacks of targeted optical imaging with near-infrared fluorescent tracers. Human vascular endothelial growth factor (VEGF) was labeled with the fluorescent dye Cy5.5 (emission maximum 696 nm) for application in an *in vivo* tumor model [111]. The elevated contrast, observed in the tumor following tracer administration was assigned to elevated VEGFR expression on tumor cells and adjacent endothelia. Unfortunately, the question remained unanswered, how specific this accumulation was. The authors missed to provide data on essential parameters, including whole body distribution of the tracer or an unspecific control of equal size and distribution. Importantly, biodistribution studies and blocking experiments are a crucial requirement for such imaging studies in order to convincingly show that tracer-mediated fluorescence in fact is a reliable measure of tracer to target binding.

Mostly due to restricted penetration depth of light and strong scatter, clinical translation of optical imaging techniques seems to be limited to lesions in or close to the skin, lesions accessible by endoscopy (e.g. colon polyps), or intraoperative applications. To solve some of these limitations, current technical developments in optical imaging focus on scanners with improved penetration depth and sensitivity, including photoacoustic imaging systems as well as hybrid fluorescence molecular tomography x-ray computed tomography scanners (FMT-XCT) [112-114]. The application of either or both technologies will help to provide significant insights into the molecular mechanisms of radiation and tumor biology in vivo. However, even state of the art optical imaging is a valuable modality for preclinical imaging in small animals, allowing for the non-invasive characterization of cells and tissues on a molecular level. The use of highly specific tracers or reporter gene-based bioluminescence imaging systems will help to gain a better understanding of the molecular processes, which take place in tumors in response to irradiation and/or targeted therapy, and will finally result in a more efficient pre-therapeutic stratification of tumors for multimodal therapy.

### Positron emission tomography (PET)/single photon emission computed tomography (SPECT)

PET is a non-invasive imaging technique that visualizes the distribution and accumulation of positron-emitting tracers in the whole body with high sensitivity providing functional and molecular information on tissues. With its high sensitivity for radiotracers even in picomolar amounts, PET allows for excellent depiction of specific metabolic activity, molecules and receptors *in vivo*. Besides the established ^18^ F-fluordesoxyglucose (FDG), a broad spectrum of PET tracers is under development for the non-invasive imaging of cellular processes such as angiogenesis, proliferation and hypoxia (Table 1). DOTA-TATE has been shown to be a valuable tracer for sensitive imaging of somatostatin-receptor expression in neuroendocrine tumors and meningioma delineation [115,116]. SPECT resembles PET in its application of radioactive tracers and the detection of gamma rays for image acquisition. However, in SPECT the gamma radiation emitted by the tracer is directly measured, whereas in PET positrons emitted by the applied tracers annihilate with surrounding electrons, causing two gamma photons to be emitted in 180° directions. PET scanners detect photon emissions coincident in time, thereby providing more spatial information of the observed radiation event and, thus, higher image resolution. Different radiotracers can be applied in SPECT for functional imaging of the brain (^99m^Tc-(HMPAO) hexamethylpropylene amine oxime), the myocardium (^99m^Tc-tetrofosmin, ^99m^Tc-sestamibi), malignant (^123^I-(MIBG) metaiodobenzylguanidine), or inflammatory processes (^99m^Tc or ^111^In- *in vivo* labeled leukocytes). However, both functional molecular imaging techniques provide only limited spatial resolution and require complementary morphologic imaging for anatomic mapping and morphologic correlation.

**Table 1:** **Novel PET tracers for research and patient care beyond **^**18**^ **F-fluor-desoxyglucose (**^**18**^ **F-FDG) aim at molecular targets such as integrins and somatostatin receptors or a sensitive for up-regulated amino acid turnover or cell membrane synthesis of tumor cells**

**Nuclide**	**Tracer**	**Metabolism**	**Comment**
^11^C	^11^C - Methionine	Amino acid	Increased amino acid uptake and turnover of tumor cells
	^11^C -Tyrosine	Amino acid	
	^11^C - Leucine	Amino acid	
^18^ F	^18^ F - Fluoruracile	Amino acid	
	^18^ F - Fluorethyltyrosine	Amino acid derivative	Longer half-life of ^18^ F allows for application of tracers in imaging centers without cyclotron
	(^18^ F - FET)		
	^18^ F - Fluordesoxyglucose	Glucose metabolism	
	(^18^ F – FDG)		
	^18^ F –Fluorazomyzinarabinoside	Hypoxia	
	(^18^ F – FAZA)		
	^18^ F – Fluoromisonidazole	Hypoxia	
	(^18^ F – FMISO)		
	^18^ F – Fluorthymidine	Proliferation	
	(^18^ F – FLT)		
	^18^ F - Galacto-RGD	α_v_β_3_-Integrins	
	[^18^ F- Arg-Gly-Asp (RGD) Peptide]		
	^18^ F - Choline	Prostate carcinoma	
	^18^ F - Fluoride	Bone metabolism	
^68^Ga	^68^Ga-DOTATOC/DOTA-TATE	Somatostatin receptors (SSR)	SSR-overexpressing tumors

In morphologic imaging, particularly tomographic modalities such as CT and MRI underwent tremendous innovation during the last 15 years currently providing excellent spatial resolution, 3D imaging and increasingly also functional information from tissue perfusion and diffusion. To combine morphological and functional/molecular information in diagnostic decision making, hybrid imaging with PET/CT has entered clinical routine in oncologic imaging using mostly ^18^ F-fluordesoxyglucose (FDG) for cancer diagnosis, as predictive imaging biomarker [117], for monitoring of therapy response and radiotherapy planning [118-120]. As a highly sensitive imaging modality based on molecular biology, PET has the ability to assess functional and molecular processes in benign and malignant tissues, which are altered in the earliest stages of virtually all diseases, before morphological changes occur. It compares normal and abnormal tissues on a functional rather than morphological level as MRI and CT. Functional and molecular imaging techniques such as ^18^ F-FDG-PET/CT can be applied to define a metabolically active biological tumor volume (BTV) for radiation therapy planning [121,122], still limited by its lack of spatial resolution and relatively low specificity to reliably delineate the tumor as accurately as required by precision RT techniques like intensity-modulated radiotherapy (IMRT). ^18^ F-FDG-PET/CT has the potential to safely decrease radiotherapy volumes by better delineation of tumor and better lymph node detection [123,124] and may be used as predictive/prognostic marker [125,126]. It enables radiation dose escalation [127], and experimentally permits the definition of regions in heterogeneous tumor at greatest risk of recurrence, thus facilitating the redistribution of radiation doses within the tumor to focus on these regions – a principle which is called dose-painting by contours (DPBC) [128]. Another method of specific dose escalation in a PET-positive area is dose painting by numbers (DPBN), where an inhomogeneous radiation dose distribution is intended on a voxel-by-voxel base [129]. Recent developments in PET reconstruction focusing on time-of-flight (TOF) and point spread function (PSF) modeling bear the potential for further improvements in diagnostic performance, as shown by Schaefferkoetter and colleagues [130]. They investigated four different reconstruction schemes on real tumor patient images and found that the application of TOF and PSF modeling may help to optimize particularly the detection of small, low-intensity, focal disease in larger patients.

However, FDG is not a tumor-specific tracer and accumulation in benign lesions, such as regions of inflammation, causes false-positive results with consecutively low specificity [131]. Therefore, novel alternative tracers with higher specificity are under investigation (Table 1), including radiolabeled amino acids for monitoring protein synthesis and radiolabeled choline for monitoring cell membrane synthesis, which may allow for a dedicated characterization of the tumor microenvironment on a molecular level prior to as well as during RT and especially useful in radiotherapy planning, e.g. in high-risk prostate cancer [132]. The amino acid methionine has been used for grading, prognostication and tumor extent delineation for RT planning and showed promising results in the detection and delineation of viable tumors particularly in low-grade gliomas [133]. In clinical practice however, ^18^ F-labeled PET molecules have revealed advantages compared to those that are ^11^C-labeled due to a longer physical half-life of 110 min vs. 20 min. In this regard, ^18^ F-labeled O–(2) fluoroethyl-L-tyrosine ([^18^ F]-FET) is one of the most widely used amino acid tracers [134]. Available data suggests that for RT planning the additional use of [^18^ F]-FET-PET to conventional imaging might improve gross tumor volume delineation [135,136]. For PET-based imaging of tumor hypoxia, tracers such as ^18^ F-fluoromisonidazole (^18^ F-FMISO) and ^124^I-iodoazomycin galactopyranoside (^124^I-IAZG) were investigated by Riedl and colleagues in rats bearing liver tumors with peritoneal metastasis by dynamic microPET imaging. The authors demonstrated that ^18^ F-FMISO and ^124^I-IAZG localized the same tumor regions to be hypoxic, however with superior diagnostic quality of ^18^ F-FMISO images in the investigated Morris hepatoma model due to higher count statistics of ^18^ F-FMISO. Clinically, Thorwarth and colleagues investigated reoxygenation dynamics and its relationship to local control after radiotherapy in a small group of head-and-neck cancer patients (n = 10), based on repeated dynamic ^18^ F-FMISO PET examinations. The authors reported that a tumor control probability model was developed based on repeated ^18^ F -FMISO PET scans during RT to estimate reoxygenation time which may be applicable for hypoxia image-guided dose escalation in RT [137].

Recently, first hybrid MRI/PET scanners have been installed for patient care combining the excellent soft tissue contrast of MRI with the options of PET in functional and molecular imaging. Compared to CT, MRI provides superior soft tissue contrast together with options for perfusion, diffusion and spectroscopic imaging, as complementing functional parameters, without the use of ionizing radiation [50,138]. The combination of both imaging modalities therefore provides strong synergies for imaging physiological and pathophysiological processes *in vivo* following multiparametric morphological, functional and molecular imaging concepts in oncology, neurology and cardiology.

Accurate delineation of gross tumor volume is a prerequisite for a successful treatment of cancer with radiotherapy. FDG-PET plays an increasingly important role in radiotherapy that goes beyond staging and selection of patients. For some tumors, such as NSCLC, FDG-PET has led to the safe decrease of radiotherapy volumes, enabling radiation dose escalation and redistribution of radiation doses within the tumor (tumor heterogeneity), along with a significant role in monitoring radiotherapy response. In esophageal cancer and bronchial cancer, FDG-PET/CT has gained significant predictive importance in multimodal treatment settings particularly before, during and after neo-adjuvant radio-chemotherapy and is very helpful in target volume delineation [139,140]. Currently, besides for staging/re-staging purposes, PET/CT is playing a complementary role to other modalities such as CT and MRI for target volume delineation in radiotherapy. Standardized protocols should be established to better define what role PET and/or PET/CT scans should play in radiotherapy planning.

## Conclusions

Advances in the understanding of the pathophysiology of cancer have triggered profound developments in multimodality treatment concepts comprising surgery, radiotherapy and molecularly targeted anti-cancer agents. These novel concepts of personalized cancer therapy require careful pre-treatment stratification and timely and efficient therapy monitoring to maximize patient benefit on an individual basis. Therefore, different functional and molecular imaging methods with corresponding biomarkers are currently being developed and characterized pre-clinically with the perspective of clinical translation. Molecularly tailored adaption of MRI, CT, ultrasound, PET/CT (-MRI) and optical imaging modalities represent promising approaches for the demands of targeted combination therapy in radiation oncology.

## Competing interests

The authors declare that they have no competing interests.

## Authors’ contributions

CC: review concept, literature research and manuscript drafting, MRI and CT section. PMP: literature research and manuscript drafting ultrasound section. ME: literature research and manuscript drafting, Optical Imaging section. DAC: literature research and manuscript drafting, ultrasound section. CR: literature research and manuscript drafting, PET section. KN: literature research and manuscript drafting, MRI and CT section. KL: review concept, literature research and manuscript drafting. FW: review concept, literature research and manuscript drafting. DH: review concept, literature research and manuscript drafting. MFR: review concept and manuscript drafting. CB: review concept, literature research and manuscript drafting. MN: review concept, literature research and manuscript drafting. All authors read and approved the final manuscript.

## References

[B1] Hanahan D Weinberg RA Hallmarks of cancer: the next generation Cell 2011 144 646 674 10.1016/j.cell.2011.02.013 21376230

[B2] Van Elmpt W Pottgen C De Ruysscher D Therapy response assessment in radiotherapy of lung cancer Q J Nucl Med Mol Imaging 2011 55 648 654 22231584

[B3] Humm JL Dewhirst MW Bhujwalla ZM Introduction to the special issue on molecular imaging in radiation biology Radiat Res 2012 177 329 330 10.1667/RR2959.1 22332930

[B4] Mangoni M Vozenin MC Biti G Deutsch E Normal tissues toxicities triggered by combined anti-angiogenic and radiation therapies: hurdles might be ahead Br J Cancer 2012 107 2 308 314 10.1038/bjc.2012.236 22691970 PMC3394974

[B5] Winkelmann CT Figueroa SD Sieckman GL Rold TL Hoffman TJ Non-invasive MicroCT imaging characterization and in vivo targeting of BB2 receptor expression of a PC-3 bone metastasis model Mol Imaging Biol 2012 14 6 667 675 10.1007/s11307-012-0540-8 22314281

[B6] Boll H Nittka S Doyon F Neumaier M Marx A Kramer M Groden C Brockmann MA Micro-CT based experimental liver imaging using a nanoparticulate contrast agent: a longitudinal study in mice PLoS One 2011 6 e25692 10.1371/journal.pone.0025692 21984939 PMC3184160

[B7] Cyran CC von Einem JC Paprottka PM Schwarz B Ingrisch M Dietrich O Hinkel R Bruns CJ Clevert DA Eschbach R Dynamic contrast-enhanced computed tomography imaging biomarkers correlated with immunohistochemistry for monitoring the effects of sorafenib on experimental prostate carcinomas Invest Radiol 2012 47 49 57 10.1097/RLI.0b013e3182300fe4 21934514

[B8] Lazanyi KS Abramyuk A Wolf G Tokalov S Zophel K Appold S Herrmann T Baumann M Abolmaali N Usefulness of dynamic contrast enhanced computed tomography in patients with non-small-cell lung cancer scheduled for radiation therapy Lung Cancer 2010 70 3 280 285 10.1016/j.lungcan.2010.03.004 20371133

[B9] Miles KA Perfusion CT for the assessment of tumour vascularity: which protocol? Br J Radiol 2003 76 1 S36 S42 15456712 10.1259/bjr/18486642

[B10] Miles KA Charnsangavej C Lee FT Fishman EK Horton K Lee TY Application of CT in the investigation of angiogenesis in oncology Acad Radiol 2000 7 840 850 10.1016/S1076-6332(00)80632-7 11048881

[B11] Miles KA Griffiths MR Perfusion CT: a worthwhile enhancement? Br J Radiol 2003 76 220 231 10.1259/bjr/13564625 12711641

[B12] Tateishi U Kusumoto M Nishihara H Nagashima K Morikawa T Moriyama N Contrast-enhanced dynamic computed tomography for the evaluation of tumor angiogenesis in patients with lung carcinoma Cancer 2002 95 835 842 10.1002/cncr.10730 12209728

[B13] Lind JS Meijerink MR Dingemans AM van Kuijk C Ollers MC de Ruysscher D Postmus PE Smit EF Dynamic contrast-enhanced CT in patients treated with sorafenib and erlotinib for non-small cell lung cancer: a new method of monitoring treatment? Eur Radiol 2010 20 2890 2898 10.1007/s00330-010-1869-5 20625738 PMC2978316

[B14] Petralia G Bonello L Viotti S Preda L D’Andrea G Bellomi M CT perfusion in oncology: how to do it Cancer Imaging 2010 10 8 19 20159664 10.1102/1470-7330.2010.0001 PMC2842179

[B15] Hermans R Meijerink M Van den Bogaert W Rijnders A Weltens C Lambin P Tumor perfusion rate determined noninvasively by dynamic computed tomography predicts outcome in head-and-neck cancer after radiotherapy Int J Radiat Oncol Biol Phys 2003 57 1351 1356 10.1016/S0360-3016(03)00764-8 14630273

[B16] Zhang LJ Zhao YE Wu SY Yeh BM Zhou CS Hu XB Hu QJ Lu GM Pulmonary embolism detection with dual-energy CT: experimental study of dual-source CT in rabbits Radiology 2009 252 61 70 10.1148/radiol.2521081682 19561250

[B17] Zhang LJ Yang GF Zhao YE Zhou CS Lu GM Detection of pulmonary embolism using dual-energy computed tomography and correlation with cardiovascular measurements: a preliminary study Acta Radiol 2009 50 892 901 10.1080/02841850903095393 19639470

[B18] Zhang LJ Wu S Wang M Lu L Chen B Jin L Wang J Larson AC Lu GM Quantitative dual energy CT measurements in rabbit VX2 liver tumors: Comparison to perfusion CT measurements and histopathological findings Eur J Radiol 2012 81 1766 1775 10.1016/j.ejrad.2011.06.057 21835570

[B19] Giusti S Buccianti P Castagna M Fruzzetti E Fattori S Castelluccio E Caramella D Bartolozzi C Preoperative rectal cancer staging with phased-array MR Radiat Oncol 2012 7 29 10.1186/1748-717X-7-29 22390136 PMC3310712

[B20] Champ CE Siglin J Mishra MV Shen X Werner-Wasik M Andrews DW Mayekar SU Liu H Shi W Evaluating changes in radiation treatment volumes from post-operative to same-day planning MRI in High-grade gliomas Radiat Oncol 2012 7 220 10.1186/1748-717X-7-220 23259933 PMC3552717

[B21] Cyran CC Paprottka PM Schwarz B Sourbron S Ingrisch M von Einem J Pietsch H Dietrich O Hinkel R Bruns CJ Perfusion MRI for monitoring the effect of sorafenib on experimental prostate carcinoma: a validation study AJR Am J Roentgenol 2012 198 384 391 10.2214/AJR.11.6951 22268182

[B22] Henzler T Konstandin S Schmid-Bindert G Apfaltrer P Haneder S Wenz F Schad L Manegold C Schoenberg SO Fink C Imaging of tumor viability in lung cancer: initial results using 23Na-MRI Fortschr Geb Rontgenstr Nuklearmed 2012 184 340 344 10.1055/s-0031-1299277 22351502

[B23] Bottomley PA Spatial localization in NMR spectroscopy in vivo Ann N Y Acad Sci 1987 508 333 348 10.1111/j.1749-6632.1987.tb32915.x 3326459

[B24] Frahm J Michaelis T Merboldt KD Hanicke W Gyngell ML Chien D Bruhn H Localized NMR spectroscopy in vivo. Progress and problems NMR Biomed 1989 2 188 195 10.1002/nbm.1940020504 2641893

[B25] Robbins ME Brunso-Bechtold JK Peiffer AM Tsien CI Bailey JE Marks LB Imaging Radiation-Induced Normal Tissue Injury Radiat Res 2012 177 4 449 466 10.1667/RR2530.1 22348250 PMC3733443

[B26] Sundgren PC Cao Y Brain irradiation: effects on normal brain parenchyma and radiation injury Neuroimaging Clin N Am 2009 19 657 668 10.1016/j.nic.2009.08.014 19959011 PMC5000393

[B27] Pasantes-Morales H Franco R Torres-Marquez ME Hernandez-Fonseca K Ortega A Amino acid osmolytes in regulatory volume decrease and isovolumetric regulation in brain cells: contribution and mechanisms Cell Physiol Biochem 2000 10 361 370 10.1159/000016369 11125217

[B28] Majos C Alonso J Aguilera C Serrallonga M Acebes JJ Arus C Gili J Adult primitive neuroectodermal tumor: proton MR spectroscopic findings with possible application for differential diagnosis Radiology 2002 225 556 566 10.1148/radiol.2252011592 12409595

[B29] Stadlbauer A Gruber S Nimsky C Fahlbusch R Hammen T Buslei R Tomandl B Moser E Ganslandt O Preoperative grading of gliomas by using metabolite quantification with high-spatial-resolution proton MR spectroscopic imaging Radiology 2006 238 958 969 10.1148/radiol.2382041896 16424238

[B30] Kuroda K Suzuki Y Ishihara Y Okamoto K Temperature mapping using water proton chemical shift obtained with 3D-MRSI: feasibility in vivo Magn Reson Med 1996 35 20 29 10.1002/mrm.1910350105 8771019

[B31] Fonteyne V Villeirs G Speleers B De Neve W De Wagter C Lumen N De Meerleer G Intensity-modulated radiotherapy as primary therapy for prostate cancer: report on acute toxicity after dose escalation with simultaneous integrated boost to intraprostatic lesion Int J Radiat Oncol Biol Phys 2008 72 799 807 10.1016/j.ijrobp.2008.01.040 18407430

[B32] Roe K Mikalsen LT van der Kogel AJ Bussink J Lyng H Ree AH Marignol L Olsen DR Vascular responses to radiotherapy and androgen-deprivation therapy in experimental prostate cancer Radiat Oncol 2012 7 75 10.1186/1748-717X-7-75 22621752 PMC3441216

[B33] Sourbron S Technical aspects of MR perfusion Eur J Radiol 2010 76 304 313 10.1016/j.ejrad.2010.02.017 20363574

[B34] Franiel T Hamm B Hricak H Dynamic contrast-enhanced magnetic resonance imaging and pharmacokinetic models in prostate cancer Eur Radiol 2011 21 616 626 10.1007/s00330-010-2037-7 21184082

[B35] Nguyen VL Kooi ME Backes WH van Hoof RH Saris AE Wishaupt MC Hellenthal FA van der Geest RJ Kessels AG Schurink GW Leiner T Suitability of pharmacokinetic models for dynamic contrast-enhanced MRI of abdominal aortic aneurysm vessel wall: a comparison PLoS One 2013 8 e75173 10.1371/journal.pone.0075173 24098370 PMC3788790

[B36] Turkheimer FE Hinz R Cunningham VJ On the undecidability among kinetic models: from model selection to model averaging J Cereb Blood Flow Metab 2003 23 490 498 12679726 10.1097/01.WCB.0000050065.57184.BB

[B37] Rischke HC Schafer AO Nestle U Volegova-Neher N Henne K Benz MR Schultze-Seemann W Langer M Grosu AL Detection of local recurrent prostate cancer after radical prostatectomy in terms of salvage radiotherapy using dynamic contrast enhanced-MRI without endorectal coil Radiat Oncol 2012 7 185 10.1186/1748-717X-7-185 23114282 PMC3560084

[B38] Roe K Kakar M Seierstad T Ree AH Olsen DR Early prediction of response to radiotherapy and androgen-deprivation therapy in prostate cancer by repeated functional MRI: a preclinical study Radiat Oncol 2011 6 65 10.1186/1748-717X-6-65 21651782 PMC3130663

[B39] Bloch BN Furman-Haran E Helbich TH Lenkinski RE Degani H Kratzik C Susani M Haitel A Jaromi S Ngo L Rofsky NM Prostate cancer: accurate determination of extracapsular extension with high-spatial-resolution dynamic contrast-enhanced and T2-weighted MR imaging–initial results Radiology 2007 245 176 185 10.1148/radiol.2451061502 17717328

[B40] Fung SH Roccatagliata L Gonzalez RG Schaefer PW MR Diffusion Imaging in Ischemic Stroke Neuroimaging Clin N Am 2011 21 345 377 10.1016/j.nic.2011.03.001 21640304

[B41] Choi SH Paeng JC Sohn CH Pagsisihan JR Kim YJ Kim KG Jang JY Yun TJ Kim JH Han MH Chang KH Correlation of 18F-FDG uptake with apparent diffusion coefficient ratio measured on standard and high b value diffusion MRI in head and neck cancer J Nucl Med 2011 18 9 2579 2584 10.2967/jnumed.111.089334 21680692

[B42] Inglese M Bester M Diffusion imaging in multiple sclerosis: research and clinical implications NMR Biomed 2010 23 865 872 10.1002/nbm.1515 20882528 PMC3071990

[B43] Wybranski C Zeile M Lowenthal D Fischbach F Pech M Rohl FW Gademann G Ricke J Dudeck O Value of diffusion weighted MR imaging as an early surrogate parameter for evaluation of tumor response to high-dose-rate brachytherapy of colorectal liver metastases Radiat Oncol 2011 6 43 10.1186/1748-717X-6-43 21524305 PMC3111366

[B44] Ueno Y Kitajima K Sugimura K Kawakami F Miyake H Obara M Takahashi S Ultra-high b-value diffusion-weighted MRI for the detection of prostate cancer with 3-T MRI JMR 2013 38 1 154 160 10.1002/jmri.23953 23292979

[B45] Blackledge MD Leach MO Collins DJ Koh DM Computed diffusion-weighted MR imaging may improve tumor detection Radiology 2011 261 573 581 10.1148/radiol.11101919 21852566

[B46] Koh DM Collins DJ Orton MR Intravoxel incoherent motion in body diffusion-weighted MRI: reality and challenges AJR Am J Roentgenol 2011 196 1351 1361 10.2214/AJR.10.5515 21606299

[B47] Notohamiprodjo M Staehler M Steiner N Schwab F Sourbron SP Michaely HJ Helck AD Reiser MF Nikolaou K Combined diffusion-weighted, blood oxygen level-dependent, and dynamic contrast-enhanced MRI for characterization and differentiation of renal cell carcinoma Acad Radiol 2013 20 685 693 10.1016/j.acra.2013.01.015 23664397

[B48] Barentsz JO Richenberg J Clements R Choyke P Verma S Villeirs G Rouviere O Logager V Futterer JJ European Society of Urogenital R ESUR prostate MR guidelines 2012 Eur Radiol 2012 22 746 757 10.1007/s00330-011-2377-y 22322308 PMC3297750

[B49] Bumb A Regino CA Perkins MR Bernardo M Ogawa M Fugger L Choyke PL Dobson PJ Brechbiel MW Preparation and characterization of a magnetic and optical dual-modality molecular probe Nanotechnology 2010 21 175704 10.1088/0957-4484/21/17/175704 20368682 PMC2859998

[B50] Makowski MR Wiethoff AJ Blume U Cuello F Warley A Jansen CH Nagel E Razavi R Onthank DC Cesati RR Assessment of atherosclerotic plaque burden with an elastin-specific magnetic resonance contrast agent Nat Med 2011 17 383 388 10.1038/nm.2310 21336283

[B51] Serres S Soto MS Hamilton A McAteer MA Carbonell WS Robson MD Ansorge O Khrapitchev A Bristow C Balathasan L Molecular MRI enables early and sensitive detection of brain metastases Proc Natl Acad Sci USA 2012 109 6674 6679 10.1073/pnas.1117412109 22451897 PMC3340084

[B52] Westmeyer GG Durocher Y Jasanoff A A secreted enzyme reporter system for MRI Angew Chem Int Ed Engl 2010 49 3909 3911 10.1002/anie.200906712 20414908 PMC3065104

[B53] Bowers CR Weitekamp DP Transformation of symmetrization order to nuclear-spin magnetization by chemical reaction and nuclear magnetic resonance Phys Rev Lett 1986 57 2645 2648 10.1103/PhysRevLett.57.2645 10033824

[B54] Olsson LE Chai CM Axelsson O Karlsson M Golman K Petersson JS MR coronary angiography in pigs with intraarterial injections of a hyperpolarized 13C substance Magn Reson Med 2006 55 731 737 10.1002/mrm.20847 16538605

[B55] Svensson J Mansson S Johansson E Petersson JS Olsson LE Hyperpolarized 13C MR angiography using trueFISP Magn Reson Med 2003 50 256 262 10.1002/mrm.10530 12876701

[B56] Day SE Kettunen MI Gallagher FA Hu DE Lerche M Wolber J Golman K Ardenkjaer-Larsen JH Brindle KM Detecting tumor response to treatment using hyperpolarized 13C magnetic resonance imaging and spectroscopy Nat Med 2007 13 1382 1387 10.1038/nm1650 17965722

[B57] Kurhanewicz J Vigneron DB Brindle K Chekmenev EY Comment A Cunningham CH Deberardinis RJ Green GG Leach MO Rajan SS Analysis of cancer metabolism by imaging hyperpolarized nuclei: prospects for translation to clinical research Neoplasia 2011 13 81 97 21403835 10.1593/neo.101102 PMC3033588

[B58] Chaumeil MM Ozawa T Park I Scott K James CD Nelson SJ Ronen SM Hyperpolarized 13C MR spectroscopic imaging can be used to monitor Everolimus treatment in vivo in an orthotopic rodent model of glioblastoma Neuroimage 2012 59 193 201 10.1016/j.neuroimage.2011.07.034 21807103 PMC3196046

[B59] Day SE Kettunen MI Cherukuri MK Mitchell JB Lizak MJ Morris HD Matsumoto S Koretsky AP Brindle KM Detecting response of rat C6 glioma tumors to radiotherapy using hyperpolarized [1–13C] pyruvate and 13C magnetic resonance spectroscopic imaging Magn Reson Med 2011 65 557 563 10.1002/mrm.22698 21264939 PMC3690628

[B60] Golman K Zandt RI Lerche M Pehrson R Ardenkjaer-Larsen JH Metabolic imaging by hyperpolarized 13C magnetic resonance imaging for in vivo tumor diagnosis Cancer Res 2006 66 10855 10860 10.1158/0008-5472.CAN-06-2564 17108122

[B61] Mansson S Johansson E Magnusson P Chai CM Hansson G Petersson JS Stahlberg F Golman K 13C imaging-a new diagnostic platform Eur Radiol 2006 16 57 67 10.1007/s00330-005-2806-x 16402256

[B62] Golman K Olsson LE Axelsson O Mansson S Karlsson M Petersson JS Molecular imaging using hyperpolarized 13C Br J Radiol 2003 76 2 S118 S127 15572334 10.1259/bjr/26631666

[B63] Paprottka PM Cyran CC Zengel P von Einem J Wintersperger B Nikolaou K Reiser MF Clevert DA Non-invasive contrast enhanced ultrasound for quantitative assessment of tumor microcirculation. Contrast mixed mode examination vs. Only contrast enhanced ultrasound examination Clin Hemorheol Microcirc 2010 46 149 158 21135490 10.3233/CH-2010-1341

[B64] Willmann JK Paulmurugan R Chen K Gheysens O Rodriguez-Porcel M Lutz AM Chen IY Chen X Gambhir SS US imaging of tumor angiogenesis with microbubbles targeted to vascular endothelial growth factor receptor type 2 in mice Radiology 2008 246 508 518 10.1148/radiol.2462070536 18180339 PMC4157631

[B65] Rychak JJ Graba J Cheung AM Mystry BS Lindner JR Kerbel RS Foster FS Microultrasound molecular imaging of vascular endothelial growth factor receptor 2 in a mouse model of tumor angiogenesis Mol Imaging 2007 6 289 296 18092513

[B66] Lee DJ Lyshchik A Huamani J Hallahan DE Fleischer AC Relationship between retention of a vascular endothelial growth factor receptor 2 (VEGFR2)-targeted ultrasonographic contrast agent and the level of VEGFR2 expression in an in vivo breast cancer model J Ultrasound Med 2008 27 855 866 18499845 10.7863/jum.2008.27.6.855

[B67] Anderson CR Rychak JJ Backer M Backer J Ley K Klibanov AL scVEGF microbubble ultrasound contrast agents: a novel probe for ultrasound molecular imaging of tumor angiogenesis Invest Radiol 2010 45 579 585 10.1097/RLI.0b013e3181efd581 20733505 PMC3426362

[B68] Jun HY Park SH Kim HS Yoon KH Long residence time of ultrasound microbubbles targeted to integrin in murine tumor model Acad Radiol 2010 17 54 60 10.1016/j.acra.2009.07.017 19815430

[B69] Crouzet S Murat FJ Pommier P Poissonnier L Pasticier G Rouviere O Chapelon JY Rabilloud M Belot A Mege-Lechevallier F Locally recurrent prostate cancer after initial radiation therapy: early salvage high-intensity focused ultrasound improves oncologic outcomes Radiother Oncol 2012 105 198 202 10.1016/j.radonc.2012.09.014 23068708

[B70] Anderson CR Hu X Zhang H Tlaxca J Decleves AE Houghtaling R Sharma K Lawrence M Ferrara KW Rychak JJ Ultrasound molecular imaging of tumor angiogenesis with an integrin targeted microbubble contrast agent Invest Radiol 2011 46 215 224 10.1097/RLI.0b013e3182034fed 21343825 PMC3075480

[B71] Robinson D Liu D Steciw S Field C Daly H Saibishkumar EP Fallone G Parliament M Amanie J An evaluation of the Clarity 3D ultrasound system for prostate localization J Appl Clin Med Phys 2012 13 3753 22766945 10.1120/jacmp.v13i4.3753 PMC5716521

[B72] Guckenberger M Richter A Boda-Heggemann J Lohr F Motion compensation in radiotherapy Crit Rev Biomed Eng 2012 40 187 197 10.1615/CritRevBiomedEng.v40.i3.30 22694199

[B73] Greis C Summary of technical principles of contrast sonography and future perspectives Radiologe 2011 51 456 461 10.1007/s00117-010-2099-1 21557023

[B74] Greis C Ultrasound contrast agents as markers of vascularity and microcirculation Clin Hemorheol Microcirc 2009 43 1 9 19713597 10.3233/CH-2009-1216

[B75] Clevert DA Sommer WH Helck A Reiser M Duplex and contrast enhanced ultrasound (CEUS) in evaluation of in-stent restenosis after carotid stenting Clin Hemorheol Microcirc 2011 48 199 208 21876247 10.3233/CH-2011-1400

[B76] Clevert DA Minaifar N Kopp R Stickel M Meimarakis G Sommer W Reiser M Imaging of endoleaks after endovascular aneurysm repair (EVAR) with contrast-enhanced ultrasound (CEUS). a pictorial comparison with CTA Clin Hemorheol Microcirc 2009 41 151 168 19276513 10.3233/CH-2009-1160

[B77] Helck A Sommer WH Wessely M Notohamiprodjo M Reiser M Clevert DA Benefit of contrast enhanced ultrasound for detection of ischaemic lesions and arterio venous fistulas in renal transplants - a feasibility study Clin Hemorheol Microcirc 2011 48 149 160 21876243 10.3233/CH-2011-1398

[B78] Clevert DA Sommer WH Helck A Saam T Reiser M Improved carotid atherosclerotic plaques imaging with contrast-enhanced ultrasound (CEUS) Clin Hemorheol Microcirc 2011 48 141 148 21876242 10.3233/CH-2011-1403

[B79] Clevert DA Helck A Paprottka PM Schwarz F Reiser MF [Latest developments in ultrasound of the liver] Radiologe 2011 51 661 670 10.1007/s00117-010-2124-4 21847777

[B80] Zengel P Schrotzlmair F Kramer M Paprottka P Clevert DA [Management of salivary gland diseases with contrast-enhanced ultrasound] Radiologe 2011 51 490 496 10.1007/s00117-010-2104-8 21614648

[B81] Clevert DA Helck A Paprottka PM Reiser MF Jung EM [Contrast-enhanced ultrasound imaging of the carotid artery] Radiologe 2011 51 483 489 10.1007/s00117-010-2102-x 21584857

[B82] Jung EM Uller W Stroszczynski C Clevert DA Contrast-enhanced sonography. Therapy control of radiofrequency ablation and transarterial chemoembolization of hepatocellular carcinoma Radiologe 2011 51 462 468 10.1007/s00117-010-2101-y 21557022

[B83] Schwarz F Sommer WH Reiser M Clevert DA [Contrast-enhanced sonography for blunt force abdominal trauma] Radiologe 2011 51 475 482 10.1007/s00117-010-2103-9 21607763

[B84] Paprottka PM Zengel P Ingrisch M Cyran CC Eichhorn M Reiser MF Nikolaou K Clevert DA [Contrast-enhanced ultrasound in animal models] Radiologe 2011 51 506 513 10.1007/s00117-010-2105-7 21626179

[B85] Stieger SM Bloch SH Foreman O Wisner ER Ferrara KW Dayton PA Ultrasound assessment of angiogenesis in a matrigel model in rats Ultrasound Med Biol 2006 32 673 681 10.1016/j.ultrasmedbio.2005.12.008 16677927 PMC1636846

[B86] Lassau N Chami L Chebil M Benatsou B Bidault S Girard E Abboud G Roche A Dynamic contrast-enhanced ultrasonography (DCE-US) and anti-angiogenic treatments Discov Med 2011 11 18 24 21276407

[B87] Pieters B Wijkstra H van Herk M Kuipers R Kaljouw E de la Rosette J Koning C Contrast-enhanced ultrasound as support for prostate brachytherapy treatment planning J Contemp Brachytherapy 2012 4 69 74 23349647 10.5114/jcb.2012.29362 PMC3552627

[B88] Krix M Plathow C Essig M Herfarth K Debus J Kauczor HU Delorme S Monitoring of liver metastases after stereotactic radiotherapy using low-MI contrast-enhanced ultrasound–initial results Eur Radiol 2005 15 677 684 10.1007/s00330-004-2620-x 15729565

[B89] Greis C Quantitative evaluation of microvascular blood flow by contrast-enhanced ultrasound (CEUS) Clin Hemorheol Microcirc 2011 49 137 149 22214685 10.3233/CH-2011-1464

[B90] Korpanty G Carbon JG Grayburn PA Fleming JB Brekken RA Monitoring response to anticancer therapy by targeting microbubbles to tumor vasculature Clin Cancer Res 2007 13 323 330 10.1158/1078-0432.CCR-06-1313 17200371

[B91] Weller GE Wong MK Modzelewski RA Lu E Klibanov AL Wagner WR Villanueva FS Ultrasonic imaging of tumor angiogenesis using contrast microbubbles targeted via the tumor-binding peptide arginine-arginine-leucine Cancer Res 2005 65 533 539 15695396

[B92] Xuan JW Bygrave M Valiyeva F Moussa M Izawa JI Bauman GS Klibanov A Wang F Greenberg NM Fenster A Molecular targeted enhanced ultrasound imaging of flk1 reveals diagnosis and prognosis potential in a genetically engineered mouse prostate cancer model Mol Imaging 2009 8 209 220 19728975

[B93] Kiessling F Fokong S Koczera P Lederle W Lammers T Ultrasound microbubbles for molecular diagnosis, therapy, and theranostics J Nucl Med 2012 53 345 348 10.2967/jnumed.111.099754 22393225

[B94] Ophir J Cespedes I Ponnekanti H Yazdi Y Li X Elastography: a quantitative method for imaging the elasticity of biological tissues Ultrason Imaging 1991 13 111 134 1858217 10.1177/016173469101300201

[B95] De Zordo T Chhem R Smekal V Feuchtner G Reindl M Fink C Faschingbauer R Jaschke W Klauser AS Real-time sonoelastography: findings in patients with symptomatic achilles tendons and comparison to healthy volunteers Ultraschall Med 2010 31 394 400 10.1055/s-0028-1109809 19946833

[B96] Krouskop TA Wheeler TM Kallel F Garra BS Hall T Elastic moduli of breast and prostate tissues under compression Ultrason Imaging 1998 20 260 274 10.1177/016173469802000403 10197347

[B97] Itoh A Ueno E Tohno E Kamma H Takahashi H Shiina T Yamakawa M Matsumura T Breast disease: clinical application of US elastography for diagnosis Radiology 2006 239 341 350 10.1148/radiol.2391041676 16484352

[B98] Lorenz A Ermert H Sommerfeld HJ Garcia-Schurmann M Senge T Philippou S Ultrasound elastography of the prostate. A new technique for tumor detection Ultraschall Med 2000 21 8 15 10.1055/s-2000-8926 10746278

[B99] Van Vledder MG Boctor EM Assumpcao LR Rivaz H Foroughi P Hager GD Hamper UM Pawlik TM Choti MA Intra-operative ultrasound elasticity imaging for monitoring of hepatic tumour thermal ablation HPB (Oxford) 2010 12 717 723 10.1111/j.1477-2574.2010.00247.x 21083798 PMC3003483

[B100] Zhang D Zhang S Wan M Wang S A fast tissue stiffness-dependent elastography for HIFU-induced lesions inspection Ultrasonics 2011 51 857 869 10.1016/j.ultras.2011.03.011 21683972

[B101] Chenot J Melodelima D N’Djin WA Souchon R Rivoire M Chapelon JY Intra-operative ultrasound hand-held strain imaging for the visualization of ablations produced in the liver with a toroidal HIFU transducer: first in vivo results Phys Med Biol 2010 55 3131 3144 10.1088/0031-9155/55/11/010 20479514 PMC2921072

[B102] Cui LG Shao JH Wang JR Bai J Zhang YZ Ultrasound elastography of ethanol-induced hepatic lesions: in vitro study Chin Med Sci J 2009 24 81 85 10.1016/S1001-9294(09)60065-1 19618603

[B103] Hoyt K Forsberg F Merritt CR Liu JB Ophir J In vivo elastographic investigation of ethanol-induced hepatic lesions Ultrasound Med Biol 2005 31 607 612 10.1016/j.ultrasmedbio.2005.01.017 15866410

[B104] Adriaenssens N Belsack D Buyl R Ruggiero L Breucq C De Mey J Lievens P Lamote J Ultrasound elastography as an objective diagnostic measurement tool for lymphoedema of the treated breast in breast cancer patients following breast conserving surgery and radiotherapy Radiol Oncol 2012 46 284 295 23412910 10.2478/v10019-012-0033-z PMC3572897

[B105] Luker GD Luker KE Optical imaging: current applications and future directions J Nucl Med 2008 49 1 4 10.2967/jnumed.108.053751 18077528

[B106] Valentini G D’Andrea C Ferrari R Pifferi A Cubeddu R Martinelli M Natoli C Ubezio P Giavazzi R In vivo measurement of vascular modulation in experimental tumors using a fluorescent contrast agent Photochem Photobiol 2008 84 1249 1256 10.1111/j.1751-1097.2008.00352.x 18422875

[B107] Park JK Jang SJ Kang SW Park S Hwang SG Kim WJ Kang JH Um HD Establishment of animal model for the analysis of cancer cell metastasis during radiotherapy Radiat Oncol 2012 7 153 10.1186/1748-717X-7-153 22963683 PMC3493326

[B108] Li W Li F Huang Q Frederick B Bao S Li CY Noninvasive imaging and quantification of epidermal growth factor receptor kinase activation in vivo Cancer Res 2008 68 4990 4997 10.1158/0008-5472.CAN-07-5984 18593895 PMC4591933

[B109] Wolf F Li W Li F Li CY Non-invasive, quantitative monitoring of hyperthermia-induced EGFR activation in xenograft tumours Int J Hyperthermia 2011 27 427 434 10.3109/02656736.2011.566593 21756040

[B110] Li W Li F Huang Q Shen J Wolf F He Y Liu X Hu YA Bedford JS Li CY Quantitative, noninvasive imaging of radiation-induced DNA double-strand breaks in vivo Cancer Res 2011 71 4130 4137 10.1158/0008-5472.CAN-10-2540 21527553 PMC3117017

[B111] Backer MV Gaynutdinov TI Patel V Bandyopadhyaya AK Thirumamagal BT Tjarks W Barth RF Claffey K Backer JM Vascular endothelial growth factor selectively targets boronated dendrimers to tumor vasculature Mol Cancer Ther 2005 4 1423 1429 10.1158/1535-7163.MCT-05-0161 16170035

[B112] Deliolanis N Lasser T Hyde D Soubret A Ripoll J Ntziachristos V Free-space fluorescence molecular tomography utilizing 360 degrees geometry projections Opt Lett 2007 32 382 384 10.1364/OL.32.000382 17356660

[B113] Ntziachristos V Tung CH Bremer C Weissleder R Fluorescence molecular tomography resolves protease activity in vivo Nat Med 2002 8 757 760 10.1038/nm729 12091907

[B114] Zavattini G Vecchi S Mitchell G Weisser U Leahy RM Pichler BJ Smith DJ Cherry SR A hyperspectral fluorescence system for 3D in vivo optical imaging Phys Med Biol 2006 51 2029 2043 10.1088/0031-9155/51/8/005 16585843

[B115] Gehler B Paulsen F Oksuz MO Hauser TK Eschmann SM Bares R Pfannenberg C Bamberg M Bartenstein P Belka C Ganswindt U [68Ga]-DOTATOC-PET/CT for meningioma IMRT treatment planning Radiat Oncol 2009 4 56 10.1186/1748-717X-4-56 19922642 PMC2785827

[B116] Combs SE Ganswindt U Foote RL Kondziolka D Tonn JC State-of-the-art treatment alternatives for base of skull meningiomas: complementing and controversial indications for neurosurgery, stereotactic and robotic based radiosurgery or modern fractionated radiation techniques Radiat Oncol 2012 7 226 10.1186/1748-717X-7-226 23273161 PMC3551826

[B117] Goldberg N Kundel Y Purim O Bernstine H Gordon N Morgenstern S Idelevich E Wasserberg N Sulkes A Groshar D Brenner B Early prediction of histopathological response of rectal tumors after one week of preoperative radiochemotherapy using 18 F-FDG PET-CT imaging. A prospective clinical study Radiat Oncol 2012 7 124 10.1186/1748-717X-7-124 22853868 PMC3447722

[B118] Zhu A Lee D Shim H Metabolic positron emission tomography imaging in cancer detection and therapy response Semin Oncol 2011 38 55 69 10.1053/j.seminoncol.2010.11.012 21362516 PMC3075495

[B119] Ford EC Herman J Yorke E Wahl RL 18 F-FDG PET/CT for image-guided and intensity-modulated radiotherapy J Nucl Med 2009 50 1655 1665 10.2967/jnumed.108.055780 19759099 PMC2899678

[B120] MacDonald SL Mulroy L Wilke DR Burrell S PET/CT aids the staging of and radiotherapy planning for early-stage extranodal natural killer/T-cell lymphoma, nasal type: a case series Radiat Oncol 2011 6 182 10.1186/1748-717X-6-182 22208903 PMC3283526

[B121] Ling CC Humm J Larson S Amols H Fuks Z Leibel S Koutcher JA Towards multidimensional radiotherapy (MD-CRT): biological imaging and biological conformality Int J Radiat Oncol Biol Phys 2000 47 551 560 10.1016/S0360-3016(00)00467-3 10837935

[B122] Vees H Casanova N Zilli T Imperiano H Ratib O Popowski Y Wang H Zaidi H Miralbell R Impact of 18 F-FDG PET/CT on target volume delineation in recurrent or residual gynaecologic carcinoma Radiat Oncol 2012 7 176 10.1186/1748-717X-7-176 23088346 PMC3494570

[B123] Tsai CS Lai CH Chang TC Yen TC Ng KK Hsueh S Lee SP Hong JH A prospective randomized trial to study the impact of pretreatment FDG-PET for cervical cancer patients with MRI-detected positive pelvic but negative para-aortic lymphadenopathy Int J Radiat Oncol Biol Phys 2010 76 477 484 10.1016/j.ijrobp.2009.02.020 19464824

[B124] Mai SK Welzel G Hermann B Wenz F Haberkorn U Dinter DJ Can the radiation dose to CT-enlarged but FDG-PET-negative inguinal lymph nodes in anal cancer be reduced? Sonderb Strahlenther Onkol 2009 185 254 259 10.1007/s00066-009-1944-5 19370429

[B125] Massaccesi M Calcagni ML Spitilli MG Cocciolillo F Pelligro F Bonomo L Valentini V Giordano A (1)(8)F-FDG PET-CT during chemo-radiotherapy in patients with non-small cell lung cancer: the early metabolic response correlates with the delivered radiation dose Radiat Oncol 2012 7 106 10.1186/1748-717X-7-106 22781363 PMC3410758

[B126] Parlak C Topkan E Onal C Reyhan M Selek U Prognostic value of gross tumor volume delineated by FDG-PET-CT based radiotherapy treatment planning in patients with locally advanced pancreatic cancer treated with chemoradiotherapy Radiat Oncol 2012 7 37 10.1186/1748-717X-7-37 22429939 PMC3354998

[B127] Wurschmidt F Petersen C Wahl A Dahle J Kretschmer M [18 F]fluoroethylcholine-PET/CT imaging for radiation treatment planning of recurrent and primary prostate cancer with dose escalation to PET/CT-positive lymph nodes Radiat Oncol 2011 6 44 10.1186/1748-717X-6-44 21529377 PMC3095991

[B128] Thorwarth D Geets X Paiusco M Physical radiotherapy treatment planning based on functional PET/CT data Radiother Oncol 2010 96 317 324 10.1016/j.radonc.2010.07.012 20673689

[B129] Rickhey M Moravek Z Eilles C Koelbl O Bogner L 18F-FET-PET-based dose painting by numbers with protons Strahlenther Onkol 2010 186 320 326 10.1007/s00066-010-2014-8 20559789

[B130] Schaefferkoetter J Casey M Townsend D El Fakhri G Clinical impact of time-of-flight and point response modeling in PET reconstructions: a lesion detection study Phys Med Biol 2013 58 1465 1478 10.1088/0031-9155/58/5/1465 23403399 PMC3616316

[B131] Strauss LG Fluorine-18 deoxyglucose and false-positive results: a major problem in the diagnostics of oncological patients Eur J Nucl Med 1996 23 1409 1415 10.1007/BF01367602 8781149

[B132] Vees H Steiner C Dipasquale G Chouiter A Zilli T Velazquez M Namy S Ratib O Buchegger F Miralbell R Target volume definition in high-risk prostate cancer patients using sentinel node SPECT/CT and 18 F-choline PET/CT Radiat Oncol 2012 7 134 10.1186/1748-717X-7-134 22873771 PMC3561224

[B133] Jacobs AH Thomas A Kracht LW Li H Dittmar C Garlip G Galldiks N Klein JC Sobesky J Hilker R 18 F-fluoro-L-thymidine and 11C-methylmethionine as markers of increased transport and proliferation in brain tumors J Nucl Med 2005 46 1948 1958 16330557

[B134] Walter F la Fougere C Belka C Niyazi M Technical Issues of [(18)F]FET-PET Imaging for Radiation Therapy Planning in Malignant Glioma Patients - A Review Frontiers Oncol 2012 2 130 10.3389/fonc.2012.00130 PMC3463828 23061046

[B135] Niyazi M Geisler J Siefert A Schwarz SB Ganswindt U Garny S Schnell O Suchorska B Kreth FW Tonn JC FET-PET for malignant glioma treatment planning Radiother Oncol 2011 99 44 48 10.1016/j.radonc.2011.03.001 21458093

[B136] Weber DC Zilli T Buchegger F Casanova N Haller G Rouzaud M Nouet P Dipasquale G Ratib O Zaidi H [(18)F]Fluoroethyltyrosine- positron emission tomography-guided radiotherapy for high-grade glioma Radiat Oncol 2008 3 44 10.1186/1748-717X-3-44 19108742 PMC2628662

[B137] Thorwarth D Eschmann SM Paulsen F Alber M A model of reoxygenation dynamics of head-and-neck tumors based on serial 18 F-fluoromisonidazole positron emission tomography investigations Int J Radiat Oncol Biol Phys 2007 68 515 521 10.1016/j.ijrobp.2006.12.037 17398015

[B138] Kim MJ Current limitations and potential breakthroughs for the early diagnosis of hepatocellular carcinoma Gut Liver 2011 5 15 21 10.5009/gnl.2011.5.1.15 21461067 PMC3065088

[B139] De Ruysscher D Wanders S van Haren E Hochstenbag M Geeraedts W Utama I Simons J Dohmen J Rhami A Buell U Selective mediastinal node irradiation based on FDG-PET scan data in patients with non-small-cell lung cancer: a prospective clinical study Int J Radiat Oncol Biol Phys 2005 62 988 994 10.1016/j.ijrobp.2004.12.019 15989999

[B140] Brucher BL Weber W Bauer M Fink U Avril N Stein HJ Werner M Zimmerman F Siewert JR Schwaiger M Neoadjuvant therapy of esophageal squamous cell carcinoma: response evaluation by positron emission tomography Ann Surg 2001 233 300 309 10.1097/00000658-200103000-00002 11224616 PMC1421244

